# Supplementary material for: Distinct forms of dopamine transmission control locomotion and learning
Source: bioRxiv. 2026 Jul 25:2026.07.24.740623. Preprint. [Version 1] doi: 10.64898/2026.07.24.740623 (PMC13419766; doi:10.64898/2026.07.24.740623)
Supplement: Supplementary file 1 [file NIHPP2026.07.24.740623V1-supplement-1.pdf]

bioRxiv preprint doi: https://doi.org/10.64898/2026.07.24.740623; this version posted July 25, 2026. The copyright holder for this preprint (which was not certified by peer review) is the author/funder, who has granted bioRxiv a license to display the preprint in perpetuity. It is made available under a CC-BY 4.0 International license.

Supplemental Figure 1 (associated with Fig. 1)

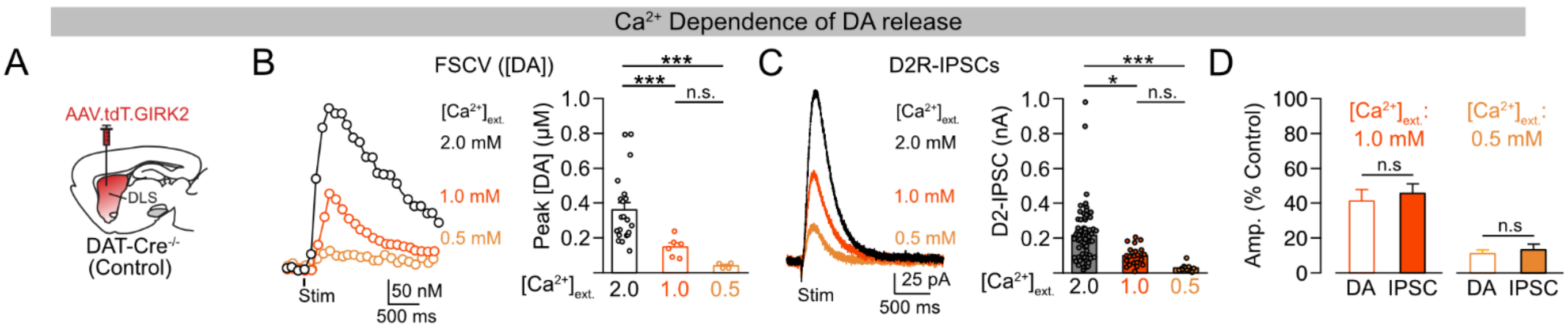

Supplemental Figure 1: Lowering extracellular [Ca^{2+}] equally reduces the amplitude of dopamine transients and D2R-IPSCs.

A. Schematic of approach to measure dopamine transients (FSCV) and D2R activation (D2R-IPSCs) by virally expressing GIRK2 channels in iSPNs of control animals.
B. Examples traces and summary of dopamine transient amplitudes in 2.0, 1.0, and 0.5 mM extracellular Ca^{2+}. Kruskal-Wallis test (*** p<0.001) followed by Tukey's test (2 mM vs. 1 mM: *** p<0.001, 2mM vs 1 mM: *** p<0.001. 1 mM vs 0.5 mM: n.s. p>0.05).
C. Examples traces and summary of D2R-IPSC amplitudes in 2.0, 1.0, and 0.5 mM extracellular Ca^{2+}. Kruskal-Wallis test followed by Tukey's test (*** p<0.001) followed by Tukey's test (2 mM vs. 1 mM * p<0.05, 2mM vs 1 mM *** p<0.001. 1 mM vs 0.5 mM n.s. p>0.05).
D. Quantification of the relative change in D2R-IPSC and dopamine transient amplitudea for 1.0 mM (n.s. p>0.05) and 0.5 mM (n.s. p>0.05) extracellular Ca^{2+}. Wilcoxon rank-sum test. Summary data are the mean ± SEM.

bioRxiv preprint doi: https://doi.org/10.64898/2026.07.24.740623; this version posted July 25, 2026. The copyright holder for this preprint (which was not certified by peer review) is the author/funder, who has granted bioRxiv a license to display the preprint in perpetuity. It is made available under a CC-BY 4.0 International license.

Supplemental Figure 2 (associated with Fig. 1)

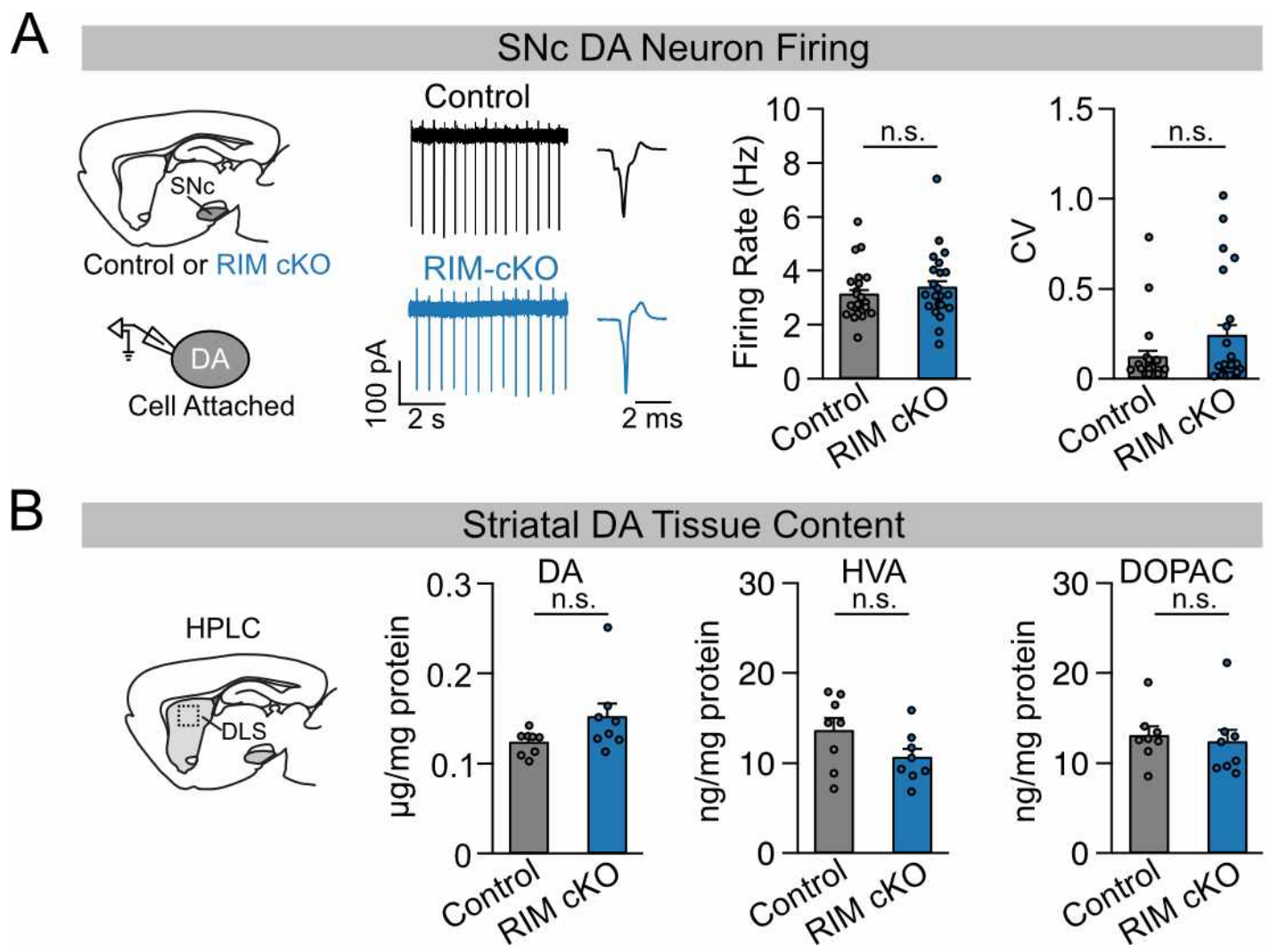

Supplemental Figure 2: Conditional knockout of RIM1/2 does not alter dopamine neuron pacemaking activity or striatal monoamine tissue content.

A. Schematic of cell-attached recording setup and examples of pacemaking activity in SNc dopamine neurons from control and RIM cKO mice (left). Quantification of intrinsic firing rate (n.s. $p>0.05$) and coefficient of variance in interspike internals (n.s. $p>0.5$) for control and RIM cKO mice (right). Wilcoxon rank-sum test.

B. Summary of dopamine (DA, n.s. $p>0.5$), homovanillic acid (HVA, n.s. p.0.5), and 3,4-dihydroxyphenylacetic acid (DOPAC, n.s. $p>0.5$) tissue content in the DLS of control and RIM cKO mice. Wilcoxon rank-sum test. Summary data are the mean ± SEM.

bioRxiv preprint doi: https://doi.org/10.64898/2026.07.24.740623; this version posted July 25, 2026. The copyright holder for this preprint (which was not certified by peer review) is the author/funder, who has granted bioRxiv a license to display the preprint in perpetuity. It is made available under a CC-BY 4.0 International license.

Supplemental Figure 3 (associated with Fig. 1)

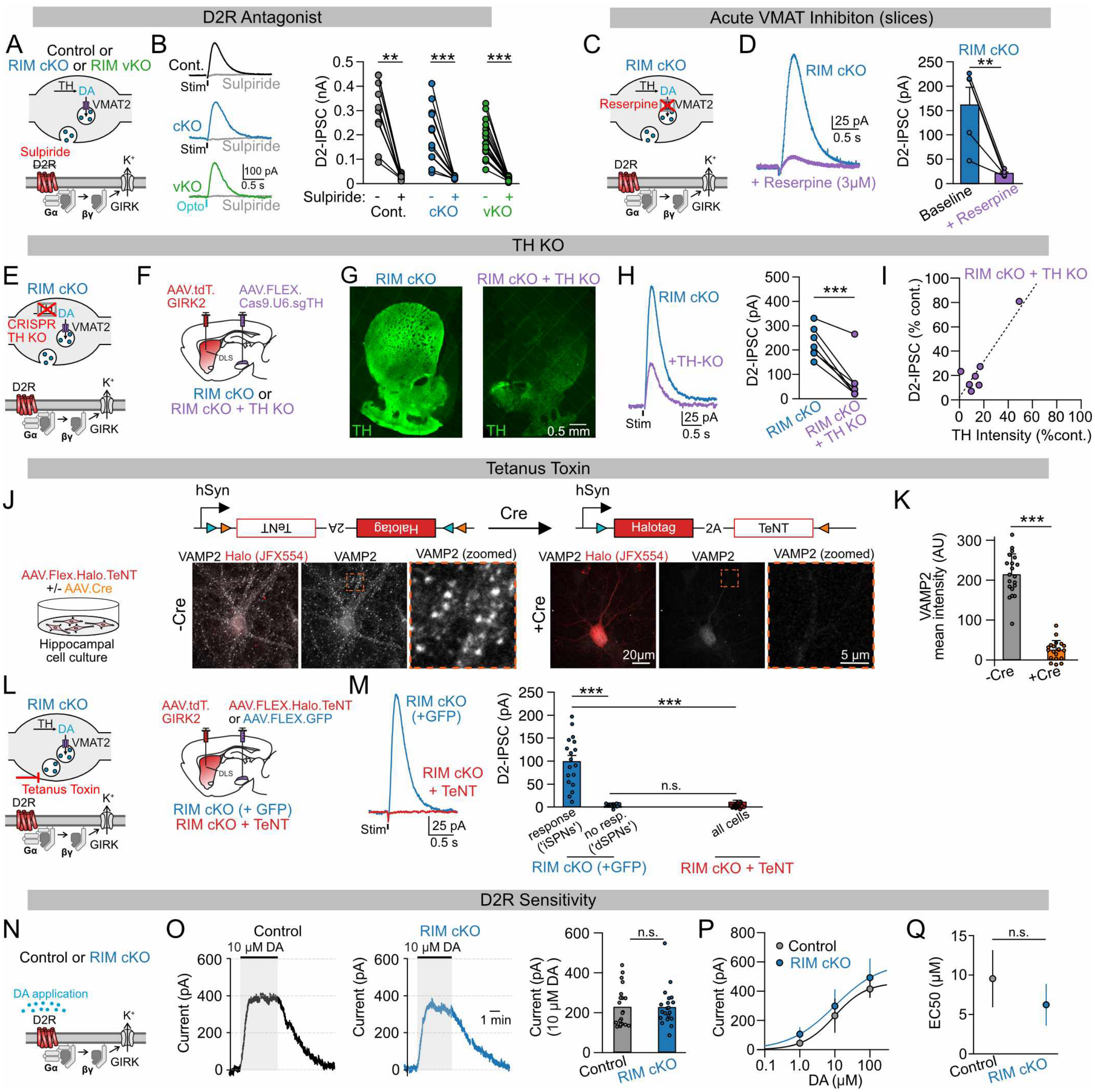

Supplemental Figure 3: Vesicular dopamine release from dopamine neurons drives D2R-IPSCs in RIM cKO mice without an appreciable upregulation D2R-mediated signaling.

A. Schematic depicting the blockade of D2Rs with the antagonist sulpiride in slices from control (Cont.), RIM cKO (cKO), or RIM vKO (cKO) mice.
B. Example traces and quantification of sulpiride's effect on D2R-IPSCs in control (** p<0.01), RIM cKO (*** p<0.001), and RIM vKO mice (*** p<0.001). Wilcoxon sign-rank.
C. Schematic depicting the blockade of the vesicular monoamine transporter 2 (VMAT2) with reserpine in slices from RIM cKO mice.
D. Example traces and quantification of reserpine's effect on D2R-IPSCs in RIM cKO mice

bioRxiv preprint doi: https://doi.org/10.64898/2026.07.24.740623; this version posted July 25, 2026. The copyright holder for this preprint (which was not certified by peer review) is the author/funder, who has granted bioRxiv a license to display the preprint in perpetuity. It is made available under a CC-BY 4.0 International license.

(*** p<0.001). Wilcoxon rank-sum test.

E. Schematic depicting CRISPR-mediated knockout of tyrosine hydroxylase (TH) in dopamine neurons.
F. Schematic of approach to measure D2R-IPSCs in RIM cKO mice with CRISPR-mediated TH knockout.
G. Example images of TH staining in the striatum of RIM cKO mice with and without CRISPR-mediated knockout of TH.
H. Examples of D2R-IPSCs in iSPNs from RIM cKO mice without (RIM cKO) and with CRISPR-mediated TH knockout (TH-KO, left). Summary of TH knockout's effect on D2R-IPSC amplitude (*** p <0.001, right). Wilcoxon sign rank test.
I. Summary of averaged normalized D2-IPSC amplitudes as a function of normalized TH staining intensity in RIM cKO mice following CRISPR-mediated TH knockout.
J. Schematic depicting Cre-dependent expression of tetanus toxin in a culture of hippocampal neurons (left). Example images of VAMP2 staining in neuronal cultures infected with virus encoding Cre-dependent tetanus toxin alone (-Cre) or coinfected with virus encoding Cre-recombinase (right, +Cre).
K. Quantification of VAMP2 staining in cultures transfected with Cre-dependent tetanus toxin alone (-Cre) or with Cre-recombinase (+Cre, ***p<0.001). Two-sided student's t-test.
L. Schematic depicting the blockade of vesicular fusion in dopamine neurons by tetanus toxin (left). Schematic showing the approach to measure D2-IPSCs in iSPNs from RIM cKO mice expressing GFP (+ GFP) or tetanus toxin (+ TeNT) in dopamine neurons.
M. Example traces of D2R-IPSCs from RIM cKO mice selectively expressing green fluorescent protein (GFP) or tetanus toxin (TeNT) in dopamine neurons (left). Quantification of D2R-IPSC amplitudes from RIM cKO mice expressing GFP (responsive and non-responsive cells) or tetanus toxin (all cells, right). Kruskal-Wallis test (*** p<0.001) followed by Tukey's post hoc test (response vs no response, *** p<0.001, response vs. TeNT, *** p<0.001, no response vs. TeNT, n.s. p>0.05).
N. Schematic of D2R-mediated GIRK currents evoked by wash-on of exogenous dopamine.
O. Example of D2R-mediate GIRK2 currents in response to 10 μM dopamine application in control and RIM cKO mice (left). Quantification of the average current amplitude for control and RIM cKO iSPNs (right, n.s. p>0.05).
P. Summary of D2R-mediated GIRK2 current amplitudes induced by exogenous application of 1, 10, or 100 μM dopamine in iSPNs from control and RIM cKO mice.
Q. Quantification of EC50 values from (P) (n.s. p>0.5). Summary data are the mean ± SEM.

bioRxiv preprint doi: https://doi.org/10.64898/2026.07.24.740623; this version posted July 25, 2026. The copyright holder for this preprint (which was not certified by peer review) is the author/funder, who has granted bioRxiv a license to display the preprint in perpetuity. It is made available under a CC-BY 4.0 International license.

Supplemental Figure 4 (associated with Fig. 1)

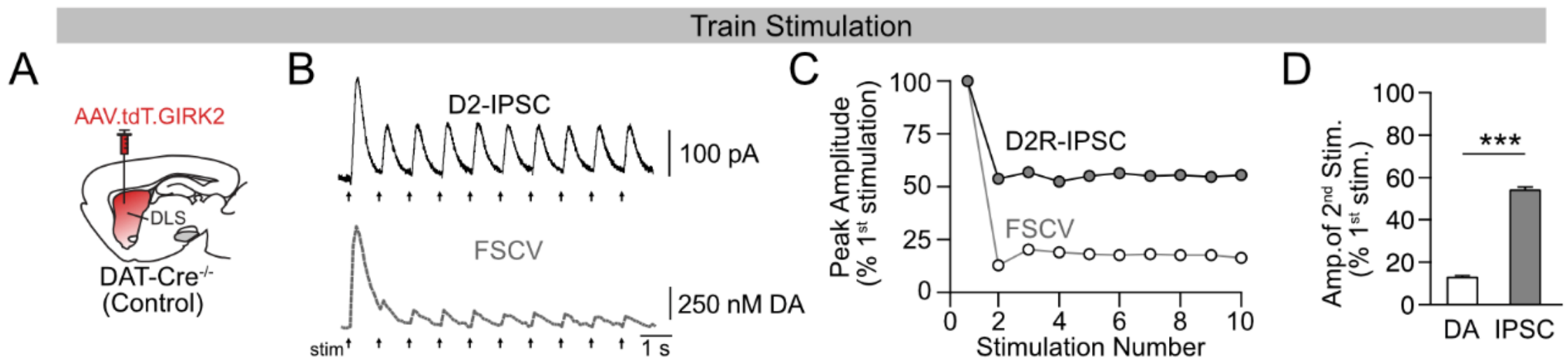

Supplemental Figure 4: Stimulus trains reveal a disconnect between spatially diffuse dopamine release and D2R activation in wild type animals.

A. Schematic of approach to measure dopamine transients (FSCV) and D2R activation (D2R-IPSCs) by virally expressing GIRK2 channels in iSPNs of control (wild type) animals.
B. Examples of D2R-IPSCs and dopamine transients evoked by a 1 Hz stimulus train.
C. Summary of D2R-IPSC and dopamine transient amplitudes normalized to the amplitude of the first response.
D. Quantification of the relative change in amplitude of dopamine transients (DA) and D2R-IPSCs (IPSC) from the first to second pulse (*** $p < 0.001$). Wilcoxon rank-sum test. Summary data are the mean ± SEM.

bioRxiv preprint doi: https://doi.org/10.64898/2026.07.24.740623; this version posted July 25, 2026. The copyright holder for this preprint (which was not certified by peer review) is the author/funder, who has granted bioRxiv a license to display the preprint in perpetuity. It is made available under a CC-BY 4.0 International license.

Supplemental Figure 5 (associated with Fig. 1)

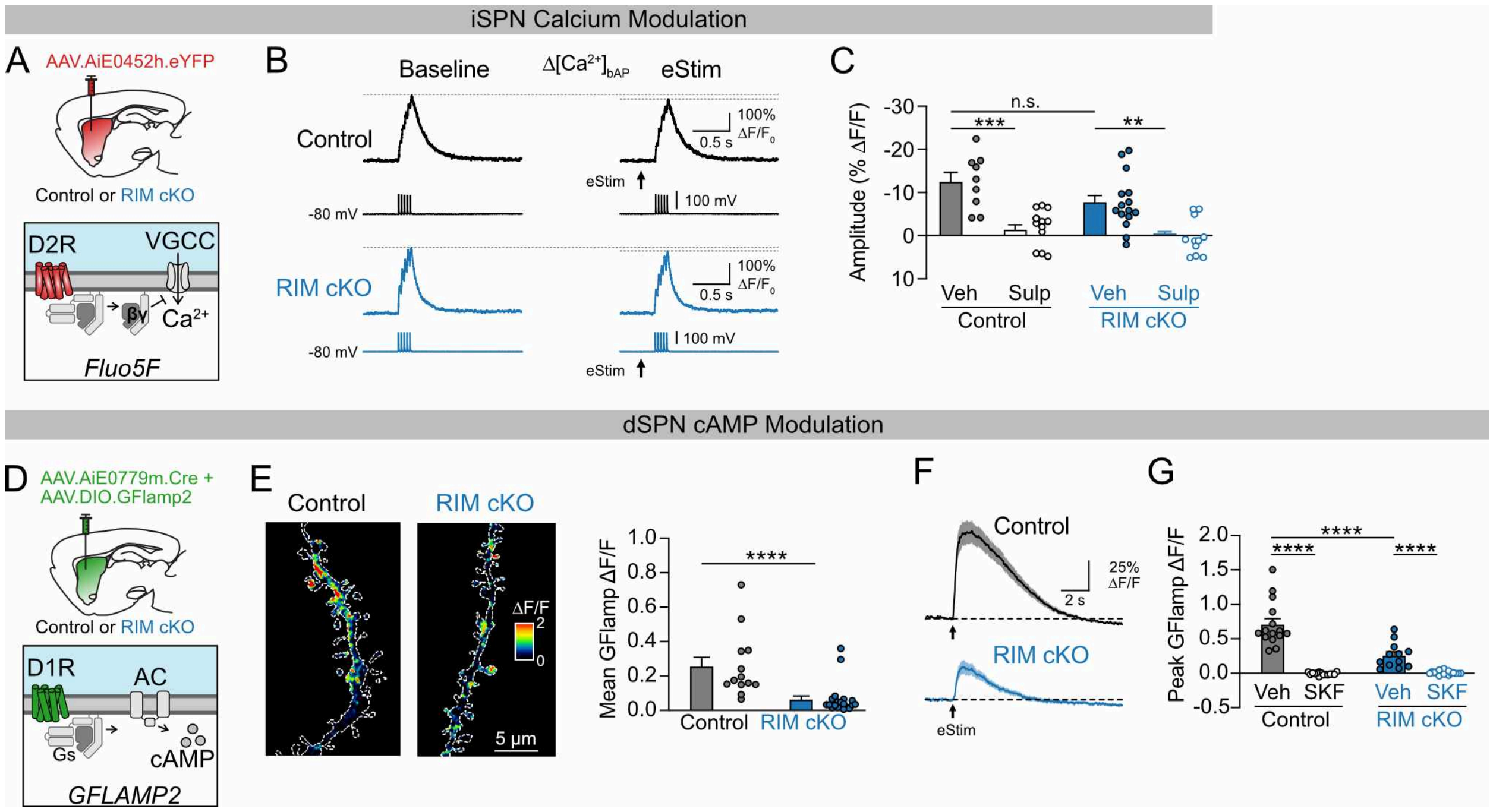

Supplemental Figure 5: Dopamine release in RIM cKO mice engages endogenous effectors downstream of D2Rs and D1Rs.

A. Schematic of experimental approach to measure D2R-mediated modulation of dendritic calcium influx induced by backpropagating action potentials in iSPNs from control and RIM cKO mice.
B. Examples of D2R-mediated reductions in dendritic calcium influx.
C. Quantification of D2R-mediated modulation of dendritic calcium influx in iSPNs from control and RIM cKO mice in the absence and presence of the D2R antagonist sulpiride. Kruskal-Wallis test (*** $p<0.001$) followed by Dunnet's post hoc test (Control Veh vs. Control Sulp: ** $p<0.001$, RIM cKO Veh vs. RIM cKO Sulp: ** $p<0.01$, Control Veh vs. RIM cKO Veh: ns $p>0.05$). (Vehicle data from Fig 1H.)
D. Schematic showing the experimental approach to measure D1R-mediated changes in dendritic cAMP dSPNs from control and RIM cKO mice with the fluorescent sensor G-Flamp2.
E. Example of GFlamp-2 activity in response to electrical stimulation in dSPNs from control and RIM cKO mice and quantification of the average change in GFlamp-2 fluorescence (ΔF/F) across dendrites in dSPNs from control and RIM cKO mice. Wilcoxon rank-sum test.
F. Example traces of GFlamp-2 activity from hotspots detected in dendrites of dSPNs from control and RIM cKO mice.
G. Quantification of hotspot amplitudes in dSPNs from control and RIM cKO mice in the absence and presence of the D1R antagonist SKF38393. Kruskal-Wallis test (*** $p<0.001$) followed by Dunnet's post hoc test (Control Veh vs. Control SKF: *** $p<0.001$, RIM cKO Veh vs. RIM cKO SKF: ** $p<0.01$, Control Veh vs. RIM cKO Veh: ns $p>0.05$). Summary data are the mean ± SEM.

bioRxiv preprint doi: https://doi.org/10.64898/2026.07.24.740623; this version posted July 25, 2026. The copyright holder for this preprint (which was not certified by peer review) is the author/funder, who has granted bioRxiv a license to display the preprint in perpetuity. It is made available under a CC-BY 4.0 International license.

Supplemental Figure 6 (associated with Fig. 2)

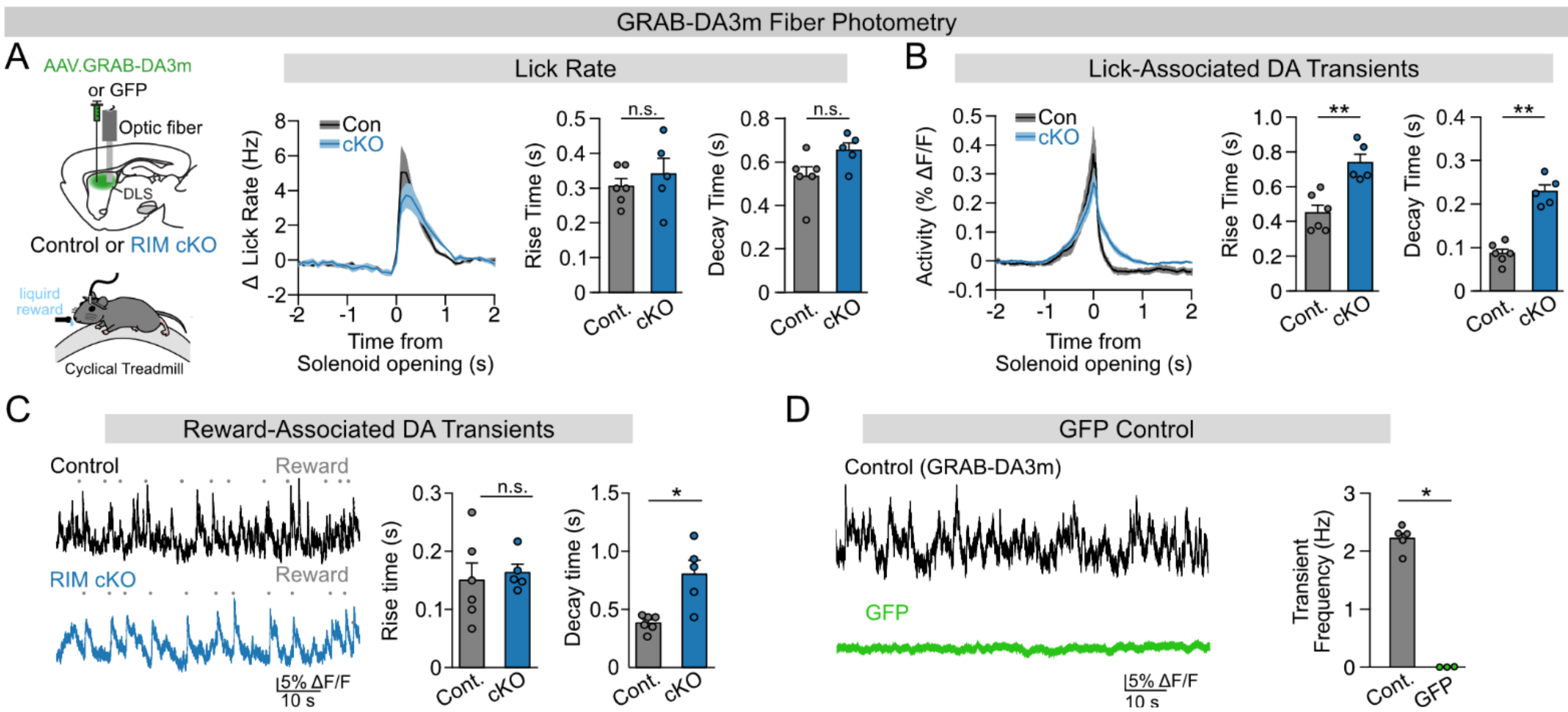

Supplemental Figure 6: RIM cKO mice show subtle effects on reward-related measures of behavior and dopamine activity.

A. Schematic depicting the approach to measure bulk dopamine release with the fluorescent sensor GRAB-DA3m in mice periodically receiving a liquid reward (left). Summary of lick rate around reward delivery (solenoid opening) in control (Cont.) and RIM cKO (cKO) mice (middle). Quantification of the time to peak (ns p>0.05) and decay time (ns p>0.05) for licking data in the middle panel (right). Wilcoxon rank-sum tests.
B. Summary of GRAB-DA3m activity (ΔF/F) aligned to licks for control and RIM cKO mice (left). Quantification of time to peak (** p<0.01) and decay time (** p<0.01) for the GRAB-DA3m data shown to the left (right). Wilcoxon rank-sum tests.
C. Example of GRAB-DA3m activity during reward delivery (grey dots) from control and RIM cKO mice (left). Quantification of rise times (n.s. p>0.05) and decay times (* p<0.05) for reward evoked transients in control (Cont.) and RIM cKO mice (cKO). Wilcoxon rank-sum tests.
D. Example photometry traces from control animals expressing GRAB-DA3m or green fluorescent protein (GFP, left). Quantification of transient frequency in mice expressing GRAB-DA3m (Cont.) and GFP (** p<0.01). Wilcoxon rank-sum test. Summary data are the mean ± SEM.

bioRxiv preprint doi: https://doi.org/10.64898/2026.07.24.740623; this version posted July 25, 2026. The copyright holder for this preprint (which was not certified by peer review) is the author/funder, who has granted bioRxiv a license to display the preprint in perpetuity. It is made available under a CC-BY 4.0 International license.

Supplemental Figure 7 (associated with Fig. 2)

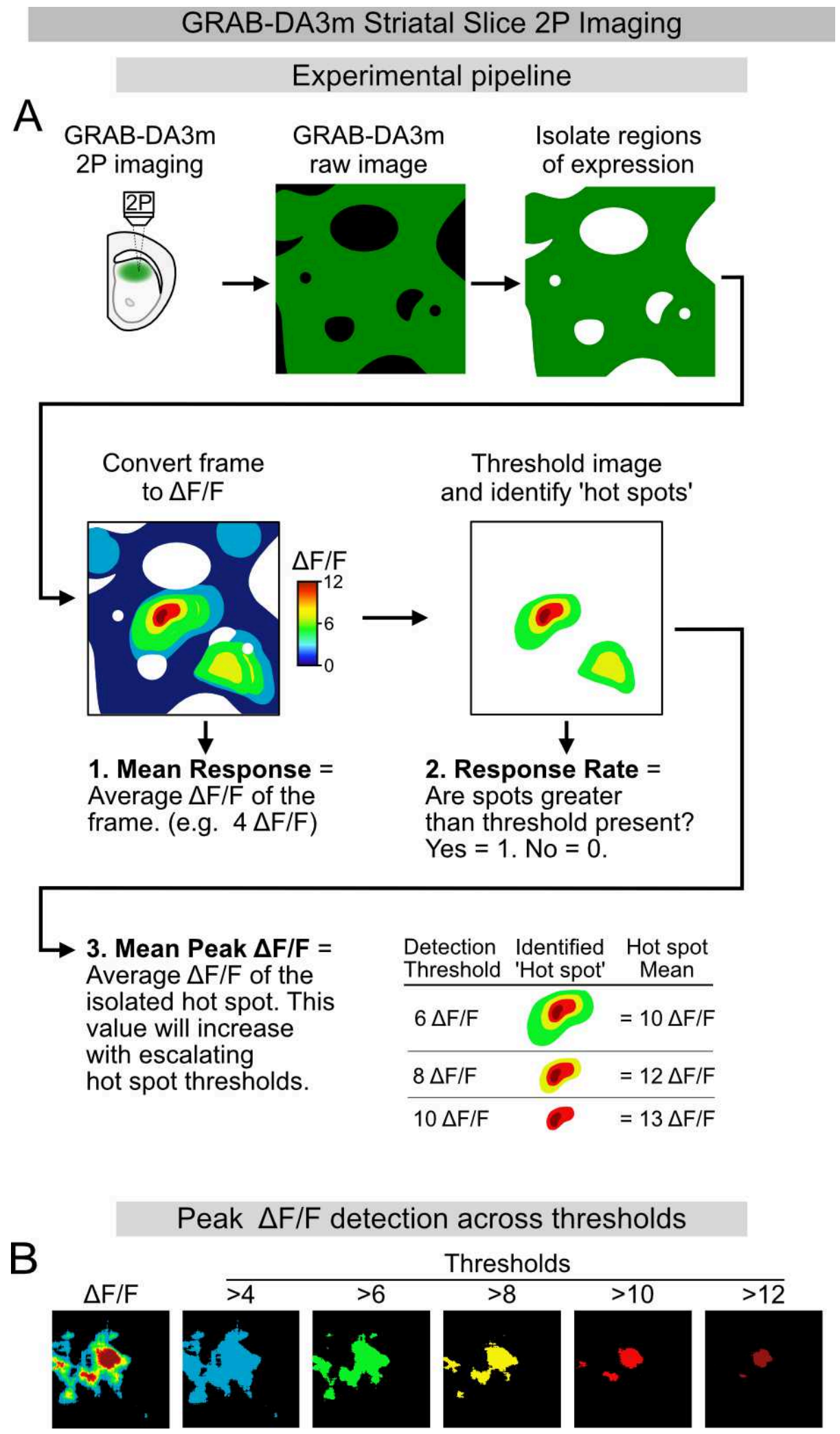

Supplemental Figure 7: Method for quantifying measures of dopamine release hotspots.

A. Schematic depicting the pipeline for detecting and analyzing hotspots of dopamine release.
B. Example of a dopamine release hotspot across a range of ΔF/F thresholds.

bioRxiv preprint doi: https://doi.org/10.64898/2026.07.24.740623; this version posted July 25, 2026. The copyright holder for this preprint (which was not certified by peer review) is the author/funder, who has granted bioRxiv a license to display the preprint in perpetuity. It is made available under a CC-BY 4.0 International license.

Supplemental Figure 8 (associated with Fig. 2)

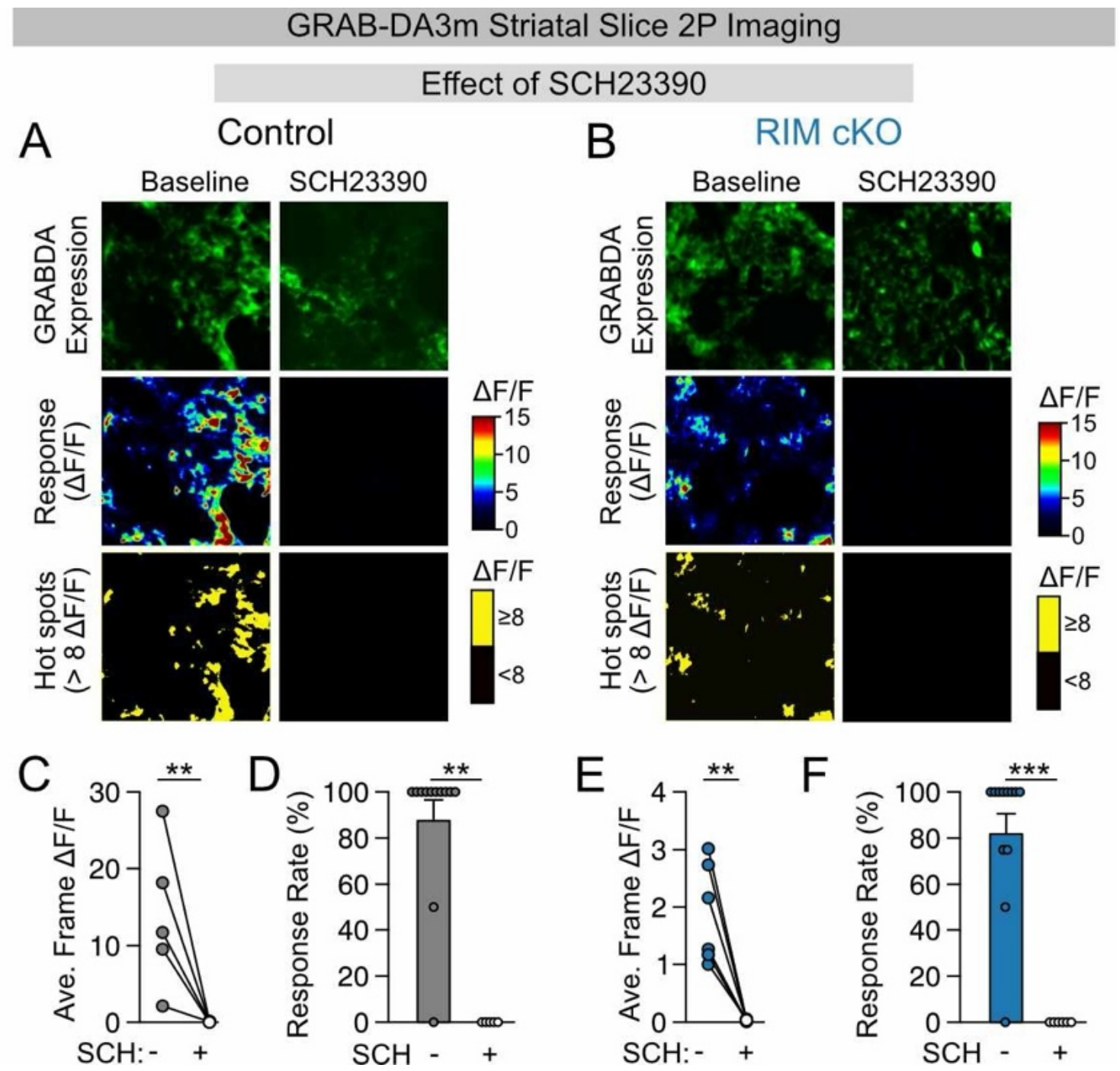

Supplemental Figure 8: The D1R antagonist SCH23390 blocks electrically evoked increase in GRAB-DA3m fluorescence and eliminates dopamine hotspots in both control and RIM cKO mice.

A. Examples of GRAB-DA3m expression, electrically evoked GRAB-DA3m activity (ΔF/F), and isolated hotspots (regions > 8 ΔF/F) in a control animal in the absence and presence of the D1R antagonist SCH23390.
B. Similar experimental approach as shown in (A), but from a RIM cKO mouse.
C. Quantification of the average GRAB-DA3m response (ΔF/F) from control mice in the absence and presence of SCH23390 (** $p<0.01$). Wilcoxon sign-rank test.
D. Quantification of response rate (presence of hotspots > 8 ΔF/F) in control mice in the absence and presence of SCH23390 (*** $p<0.001$). Wilcoxon rank-sum test.
E. Quantification of the average GRAB-DA3m response (ΔF/F) for slices from RIM cKO mice in the absence and presence of SCH23390 (** $p<0.01$). Wilcoxon sign-rank test.
F. Quantification of response rate (presence of hotspots > 8 ΔF/F) in RIM cKO mice in the absence and presence of the SCH23390 (*** $p<0.001$). Wilcoxon rank-sum test. Summary data are the mean ± SEM.

bioRxiv preprint doi: https://doi.org/10.64898/2026.07.24.740623; this version posted July 25, 2026. The copyright holder for this preprint (which was not certified by peer review) is the author/funder, who has granted bioRxiv a license to display the preprint in perpetuity. It is made available under a CC-BY 4.0 International license.

Supplemental Figure 9 (associated with Fig. 2)

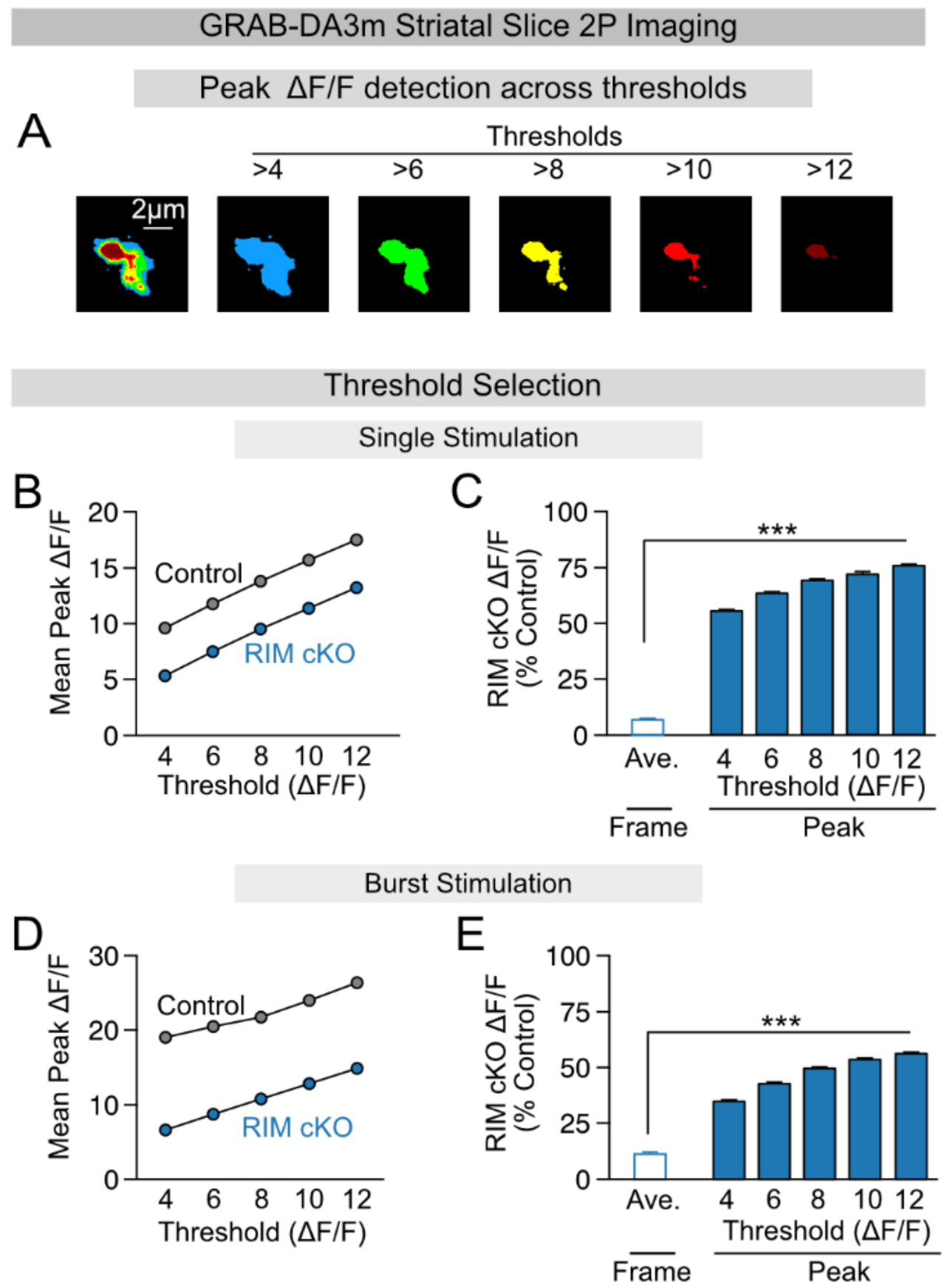

Supplemental Figure 9: The differential effect of RIM1/2 knockout on mean response and mean peak response persists across a range of ΔF/F thresholds.

A. Example of a dopamine hotspot across a range of ΔF/F thresholds.
B. Summary of the average mean amplitude within dopamine hotspots (mean peak) in response to a single electrical stimulus across a range of detection thresholds for control and RIM cKO mice.
C. Quantification of the relative reduction in mean and mean peak amplitudes across a range of detection thresholds for RIM cKO mice compared to controls. Data is for a single electrical stimulus. Kruskal-Wallis test (*** $p<0.001$) followed by Tukey's post hoc test (Ave. vs 4, 6, 8, 10, 12: *** $p<0.001$).
D. Summary of mean peak amplitudes in response to a burst stimulus (40 Hz, 5 pulse) across a range of detection thresholds for control and RIM cKO mice.
E. Quantification of the relative reduction in mean and mean peak amplitudes across a range of detection thresholds for RIM cKO mice compared to controls. Data is for a burst stimulus as described in (D). Kruskal-Wallis test (*** $p<0.001$) followed by Tukey's post hoc test (Ave. vs 4, 6, 8, 10, 12: *** $p<0.001$). Summary data are the mean ± SEM.

bioRxiv preprint doi: https://doi.org/10.64898/2026.07.24.740623; this version posted July 25, 2026. The copyright holder for this preprint (which was not certified by peer review) is the author/funder, who has granted bioRxiv a license to display the preprint in perpetuity. It is made available under a CC-BY 4.0 International license.

Supplemental Figure 10 (associated with Fig. 2)

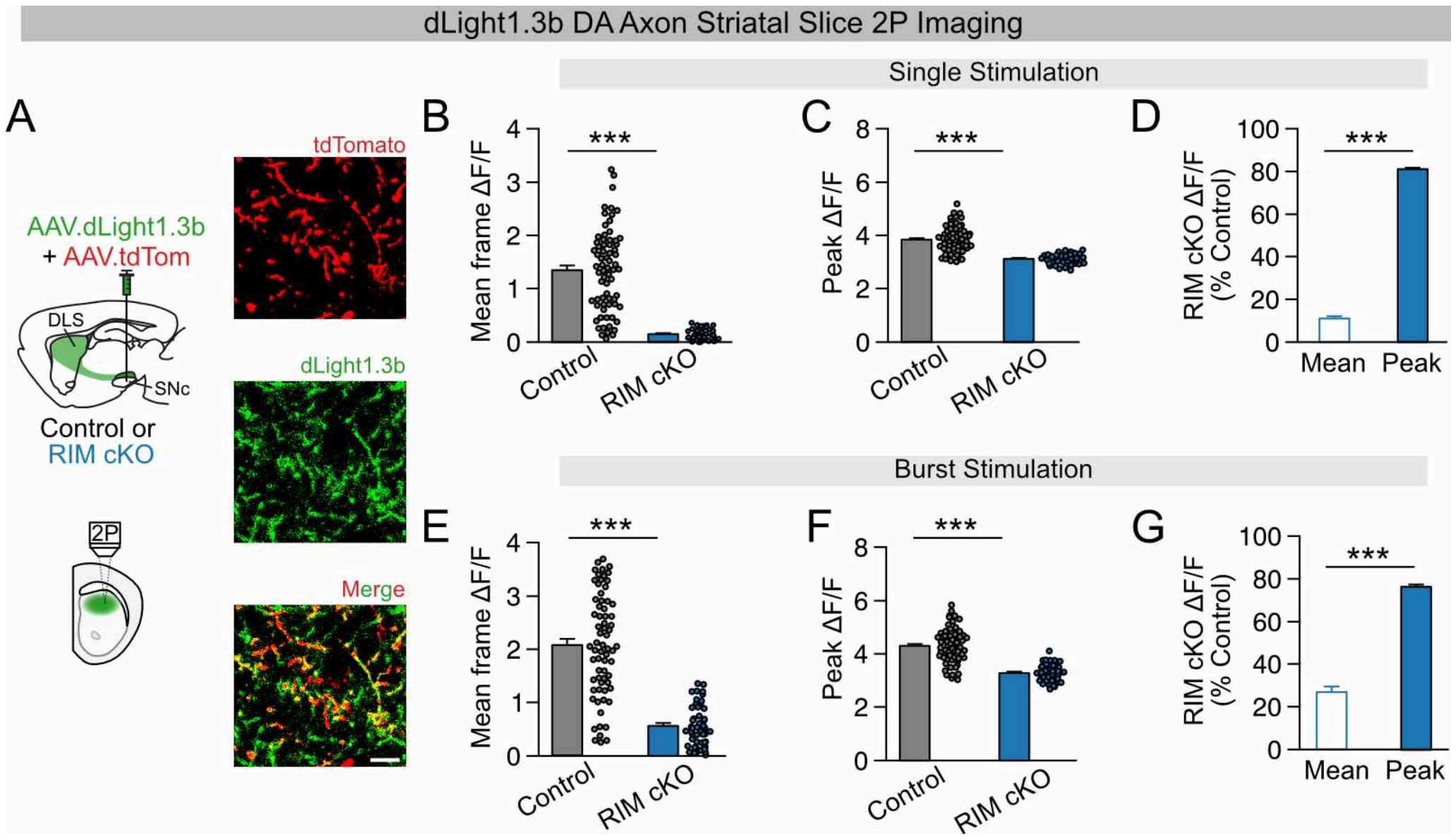

Supplemental Figure 10: Hotspots of dopamine release in RIM cKO mice are also detectable with the fluorescent dopamine sensor dLight1.3b.

A. Schematic depicting the approach to measure dopamine release in slices from mice expressing dLight1.3b and tdTomato in nigrostriatal dopamine axons (left). Example images of dopamine axons within the dorsolateral striatum labeled with dLight1.3b and tdTomato (right).
B. Quantification of the average dLight1.3b response (ΔF/F) for a single electrical stimulus in slices from control and RIM cKO mice (*** $p<0.001$). Wilcoxon rank-sum test.
C. Quantification of the average mean amplitude within dopamine hotspots (ΔF/F > 2.0) evoked by a single electrical stimulus for control and RIM cKO mice (*** $p<0.001$). Wilcoxon-rank sum test.
D. Relative decreases in average dLight1.3b response and average mean hotspot amplitude evoked by a single stimulus for RIM cKO mice compared to controls (*** $p<0.001$). Wilcoxon rank-sum test.
E. Quantification of the average dLight1.3b response (ΔF/F) to a burst stimulus (40 Hz, 5 pulses) in slices from control and RIM cKO mice (*** $p<0.001$). Wilcoxon rank-sum test.
F. Quantification of the average mean amplitude within dopamine hotspot (ΔF/F > 2.0) evoked by a burst stimulus for control and RIM cKO mice (*** $p<0.001$). Wilcoxon rank-sum test.
G. Relative decreases in average dLight1.3b response and average mean hotspot amplitude evoked by a burst stimulus for RIM cKO mice compared to controls (*** $p<0.001$). Wilcoxon rank-sum test. Summary data are the mean ± SEM.

bioRxiv preprint doi: https://doi.org/10.64898/2026.07.24.740623; this version posted July 25, 2026. The copyright holder for this preprint (which was not certified by peer review) is the author/funder, who has granted bioRxiv a license to display the preprint in perpetuity. It is made available under a CC-BY 4.0 International license.

Supplemental Figure 11 (associated with Fig. 3)

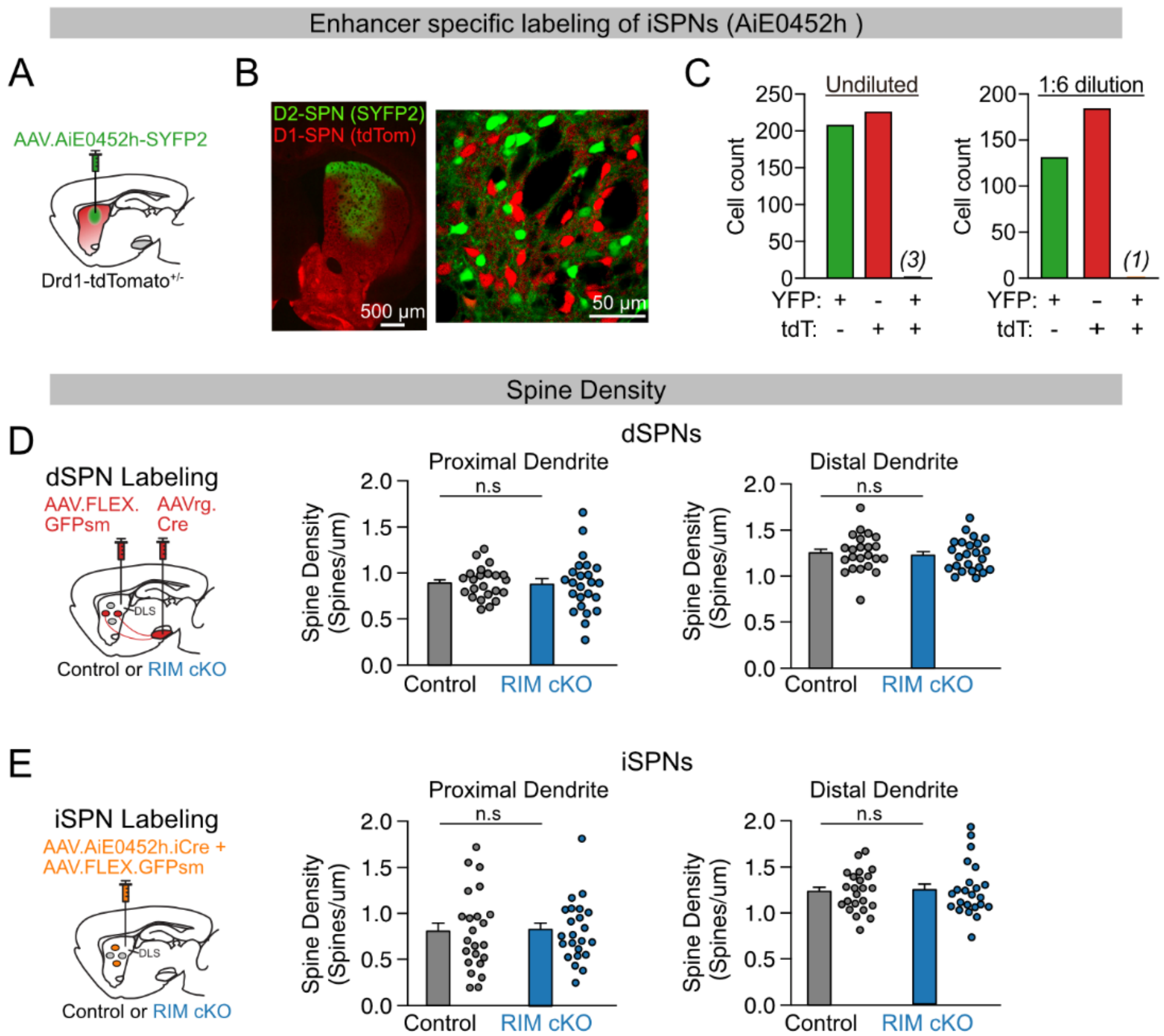

Supplemental Figure 11: Validation of viral strategy for isolating iSPNs and breakdown of SPN spine density in proximal and distal dendrites.

A. Schematic depicting the approach to label iSPNs with eYFP using an iSPN specific enhancer AAV.
B. Example image of eYFP expression in the striatum of a D1-tdTomato mouse.
C. Counts of eYFP+, tdTomato+ (tdT), and double labeled cells in mice injected with undiluted or diluted (1/6) virus.
D. Schematic showing the experimental approach for quantifying spine density in dSPNs (left). Quantification of spine density in proximal (n.s. $p>0.5$) and distal dendrites (n.s. $p>0.5$) of dSPNs from control and RIM cKO mice (right). Wilcoxon rank-sum test.
E. Schematic showing the experimental approach for quantifying spine density in iSPNs (left). Quantification of spine density in proximal (n.s. $p>0.5$) and distal dendrites (n.s. $p>0.5$) of iSPNs from control and RIM cKO mice (right). Wilcoxon rank-sum test. Summary data are the mean ± SEM.

bioRxiv preprint doi: https://doi.org/10.64898/2026.07.24.740623; this version posted July 25, 2026. The copyright holder for this preprint (which was not certified by peer review) is the author/funder, who has granted bioRxiv a license to display the preprint in perpetuity. It is made available under a CC-BY 4.0 International license.

Supplemental Figure 12 (associated with Fig. 4)

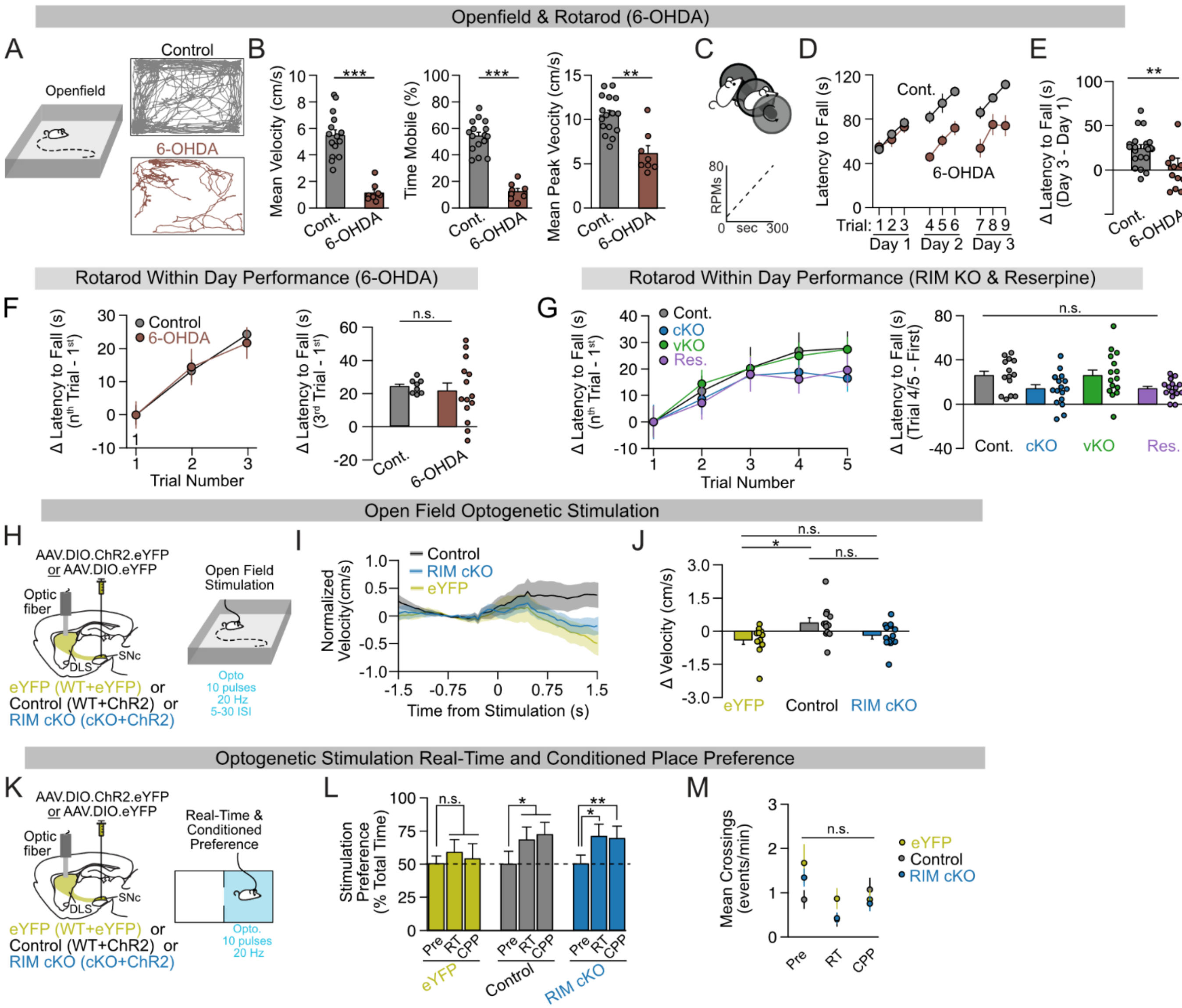

Supplemental Figure 12: Behavioral controls related to how spatially diffuse and point-to-point dopamine transmission influence motor output and motor learning.

A. Schematic of the open field assay and example position plots for a control and 6-OHDA-treated animal.
B. Quantification of mean velocity (*** p<0.001), percent time mobile (*** p<0.001), and average peak velocity (** p<0.01) for control and 6-OHDA-treated mice. Wilcoxon rank-sum test.
C. Schematic of the accelerating rotarod assay protocol.
D. Rotarod performance (latency to fall) over the course of 3 days for control and 6-OHDA-treated mice.
E. Quantification of change in performance for control and 6-OHDA-treated mice (** p<0.01). Wilcoxon rank-sum test.
F. Summary of the within day changes in performance for control and 6-OHDA-treated mice. Quantification of the changes in performance within days for each group (right, ns

bioRxiv preprint doi: https://doi.org/10.64898/2026.07.24.740623; this version posted July 25, 2026. The copyright holder for this preprint (which was not certified by peer review) is the author/funder, who has granted bioRxiv a license to display the preprint in perpetuity. It is made available under a CC-BY 4.0 International license.

p>0.05). Wilcoxon rank-sum test.

G. Within day performance changes for control (Cont.), RIM cKO (cKO), RIM vKO (vKO), and reserpine-treated (Res., 0.5 mg/kg) mice (left). Quantification of the within day changes across groups (ns p>0.05). Kruskal-Wallis test (ns p>0.05).
H. Schematic of the paradigm for optogenetically stimulating nigrostriatal dopamine axons. Groups included wild type mice expressing eYFP (Control + eYFP) or ChR2 (Control +ChR2) selectively within dopamine neurons and RIM cKO mice expressing ChR2 (RIM cKO + ChR2) within dopamine neurons.
I. Change in velocity following light deliver during periods of mobility (>2 cm/s) for the groups described in (H).
J. Quantification of the change in velocity following light delivery during periods of mobility (eYFP vs. Control, * p<0.5). Kruskal-Wallis test (* p<0.05) followed by Tukey's post hoc test (eYFP vs. Control: * p<0.05), eYFP vs. RIM cKO: ns p>0.05, Control vs. RIM cKO: ns p>0.05).
K. Experimental paradigm for optogenetic real-time and conditioned place preference.
L. Summary of preference for the light-paired chamber at baseline (pre), during real-time place preference (RT), and during conditioned place-preference test session (CPP). (eYFP Pre vs. RT: n.s. p>0.05, eYFP Pre vs. CPP: n.s. p>0.05, Control Pre vs. RT: * p<0.05, Control Pre vs. CPP: * p<0.05, RIM cKO Pre vs. RT: * p<0.05, RIM cKO Pre vs. CPP: **p<0.01). Wilcoxon rank-sum tests.
M. Quantification of the average number of crossings per minute across groups for the different test periods (ns p>0.05). Kruskal-Wallis test for each session type. Summary data are the mean ± SEM.

bioRxiv preprint doi: https://doi.org/10.64898/2026.07.24.740623; this version posted July 25, 2026. The copyright holder for this preprint (which was not certified by peer review) is the author/funder, who has granted bioRxiv a license to display the preprint in perpetuity. It is made available under a CC-BY 4.0 International license.

Table 1.1

Experiment	Virus	Site	Volume	Dilution	Provider	ID
GIRK Recordings	AAV9-Syn-tdTomato-T2A-mGIRK2	DLS	400 nL	1:3	UPenn Viral Core	V3992
Viral RIM Knockout	AAV5-Ef1a-Cre-mcherry and			1:3	UNC Viral Core	55632
	AAV5-EF1a-DIO-hChR2(H134R)-eYFP	SNc	400 nL	1:3	Addgene	20298-AAV5
TH Knockout	AAV1-CMV-FLEX-SaCas9-U6-sgTh	SNc	400 nL	1:1	Addgene	159901
Tetanus Toxin Expression	AAV5-hSyn-DIO-Halotag-2A-TeNT or	SNc	400 nL	NA	Kennedy Lab	NA
	AAV9-pCAG-FLEX-EGFP	SNc	400 nL	1:9	Addgene	51502-AAV9
6-OHDA Lesions	6-hydroxydopamine (6-OHDA)	MFB	1000 nL	4µg/µL	Sigma-Aldrich	162957
Striatal DA Imaging	AAV1-hSyn-GRAB-DA3m or	DLS	400 nL	1:3	Addgene	208698-AAV1
	AAV9-Syn-dLight1.3b	DLS	400 nL	1:7	Addgene	135762-AAV9
Axonal DA Imaging	AAV5-FLEX-CAG-dLight1.3b and			1:7	Vigene	6833
	pAAV5-CAG-tdTomato	SNc	400 nL	1:79	Addgene	59462-AAV5
Intrinsic Excitability	AAV9-AiE0452h-minBG-SYFP2	DLS	400 nL	1:6	Addgene	224173
Retrograde labeling	AAVrg-hSyn-Cre-P2A-dTomato or	Gpe	200 uL	NA	Addgene	107738-AAVrg
	AAVrg-hSyn-Cre-P2A-dTomato and	SNr	400 nL	NA	Addgene	107738-AAVrg
	AAV1-CAG-FLEX-GFPsm_myc	DLS	400 nL	1:19	Addgene	98927-AAV1
	Red Retrobeads or	Gpe	200 nL	1:3	Lumafluor	NA
	Red Retrobeads	SNr	400 nL	1:3	Lumafluor	NA
Optogenetics	AAV5-EF1a-DIO-hChR2(H134R)-eYFP	SNc	400 nL	1:3	Addgene	20298-AAV5
	or AAV5-EF1a-DIO-EYFP	SNc	400 nL	1:1	Addgene	27056-AAV5
Fiber Photometry	AAV9-hSyn-GRAB-gDA3m or	DLS	400 nL	1:10	Biohippo	PT-4720
	AAV1-CMV-PI.EGFP-WPRE-bGH	DLS	400 nL	1:10	Addgene	105530-AAV1
cAMP Imaging	pAAV-AiE0779m_3xC2-minBG-iCre(R297T) and			1:7	Addgene	199776-PHPeB
	AAV2/9-hSyn-DIO-opt-GFlamp2	DLS	400 nL	1:7	BCH Viral Core	
VGCCs Activity	AAV9-AiE0452h-minBG-SYFP2	DLS	400 nL	1:3	Addgene	224173

bioRxiv preprint doi: https://doi.org/10.64898/2026.07.24.740623; this version posted July 25, 2026. The copyright holder for this preprint (which was not certified by peer review) is the author/funder, who has granted bioRxiv a license to display the preprint in perpetuity. It is made available under a CC-BY 4.0 International license.

**Figure 1**	**Animals**	**Sample Size**	**p-values**	**Stars**	**Statistical test**
Panel B	Control N=12 6OHDA=6	Control=68 6OHDA=59	p=3.18E-22	***	Wilcoxon rank-sum
	Control N=12 6OHDA=6	Control=21 6OHDA=12	p=0.00000264	***	Wilcoxon rank-sum
Panel C	Control = 3 Reserpine=3	Control = 20 Reserpine= 20	p=0.000621	***	Wilcoxon rank-sum
	Control = 6 Reserpine=6	Control = 16 Reserpine=16	p=0.000737	***	Wilcoxon rank-sum
Panel D	Control = 12 cKO=13	Control=68 cKO=56	p=0.0428	*	Wilcoxon rank-sum
	Control = 12 cKO=13	Control =21 cKO=25	p=0.0000249	***	Wilcoxon rank-sum
Panel E	Control=6 vKO=8	Control=40 vKO=40	p=0.411	ns	Wilcoxon rank-sum
	Control=6 vKO=8	Control=15 vKO=17	p=0.0000198	***	Wilcoxon rank-sum
Panel F	6OHDA GIRK=6 6OHDA Volt=6	GIRK=59 Volt=12	p=0.369	ns	Wilcoxon rank-sum
	Res. GIRK=3 Res. Volt=6	GIRK=20 Volt=16	p=1.00	ns	Wilcoxon rank-sum
	cKO GIRK= 12 cKO Volt =12	GIRK=68 Volt=21	p=1.80E-12	***	Wilcoxon rank-sum
	vKO GIRK= 12 vKO Volt =12	GIRK=40 Volt=17	p=4.85E-09	***	Wilcoxon rank-sum
Panel H	Contrl=3 cKO=5	Control=9 cKO=15	p=0.121	ns	Wilcoxon rank-sum
**Figure 2**					
Panel C	cKO=3	cKO=3	p=0.25	ns	Signed-Rank
Panel D	Control = 4	Control=4	p=0.0625	ns	Signed-Rank
Panel E	Control = 6 cKO=5	Control = 6 cKO=5	p=0.0043	**	Wilcoxon rank-sum
	Control = 6 cKO=5	Control = 6 cKO=5	p=0.0043	**	Wilcoxon rank-sum
Panel F	Control = 6 cKO=5	Control = 6 cKO=5	p=0.0173	*	Wilcoxon rank-sum
Panel I	Control=3 cKO=3	Control=11 cKO=10	p=0.000376	***	Wilcoxon rank-sum
Panel J	Control=3 cKO=3	Control=11 cKO=10	p=0.000165	***	Wilcoxon rank-sum
Panel K	Mean cKO=3 Peak cKO=3	Mean cKO=10 Peak cKO=10	p=0.000183	***	Wilcoxon rank-sum
Panel L	Control=3 cKO=3	Control=12 cKO=12	p=0.002	**	Wilcoxon rank-sum
Panel M	Control=3 cKO=3	Control=12 cKO=12	p=0.000901	***	Wilcoxon rank-sum
Panel N	Mean cKO=3 Peak cKO=3	Mean cKO=12 Peak cKO=12	p=0.0000366	***	Wilcoxon rank-sum
Panel Q	Control=3 cKO=3	Control=11 cKO=10	p=4.318E-7	***	Kruskal-Wallis
		Shuffle vs. cKO	p=0.008524	**	Tukey's
		Shuffle vs. Control	p=1.94E-07	***	Tukey's
		cKO vs. Control	p=0.0388	*	Tukey's
**Figure 3**					
Panel C	Control=3 cKO=3	Control=22 cKO=26	p=0.001	**	Wilcoxon rank-sum
Panel D	Control=3 cKO=3	Control=22 cKO=26	p=0.0322	*	Wilcoxon rank-sum
Panel G	Control=4 cKO=4	Control=25 cKO=25	p0.00170	**	Wilcoxon rank-sum
Panel H	Control=4 cKO=4	Control=25 cKO=25	p=0.1073	ns	Wilcoxon rank-sum
Panel K	Control=3 cKO=3	Control=23 cKO=24	p=0.663	ns	Wilcoxon rank-sum
	Control=3 cKO=3	Control=23 cKO=24	p=0.558	ns	Wilcoxon rank-sum
	Control=3 cKO=3	Control=23 cKO=24	p=0.758	ns	Wilcoxon rank-sum
PanelL	Control=3 cKO=3	Control=24 cKO=29	p=0.396	ns	Wilcoxon rank-sum
	Control=3 cKO=3	Control=24 cKO=29	p=0.851	ns	Wilcoxon rank-sum
	Control=3 cKO=3	Control=24 cKO=29	p=0.514	ns	Wilcoxon rank-sum
**Figure 4**					
Panel B	Cont.=16 Res.=15 cKO=17 vKO=13	Cont.=16 Res.=15 cKO=17 vKO=13	p=0.000000644	***	Kruskal-Wallis
	Control vs. Reserpine		p=0.000000163	***	Dunnett
	Control vs cKO		p=0.0116	*	Dunnett
	Control vs vKO		p=0.000523	***	Dunnett
Panel C	Cont.=16 Res.=15 cKO=17 vKO=13	Cont.=16 Res.=15 cKO=17 vKO=13	p=0.00000112	***	Kruskal-Wallis
	Control vs. Reserpine		p=0.000000199	***	Dunnett
	Control vs cKO		p=0.0128	*	Dunnett
	Control vs vKO		p=0.00148	**	Dunnett
Panel D	Cont.=16 Res.=15 cKO=17 vKO=13	Cont=16 Res.=15 cKO=17 vKO=13	p=0.000000456	***	Kruskal-Wallis
	Control vs. Reserpine		p=0.000000201	***	Dunnett
	Control vs cKO		p=0.0835	ns	Dunnett
	Control vs vKO		p=0.000625	***	Dunnett
Panel G	Cont.=14 Res.=14 cKO=17 vKO=16	Cont.=14 Res.=14 cKO=17 vKO=16	p=0.000768	***	Kruskal-Wallis
	Control vs. Reserpine	Control=14 Reserpine=14	p=0.000762	***	Dunnett
	Control vs cKO	Control=14 cKO=17	p=0.681	ns	Dunnett
	Control vs vKO	Control=14 cKO=16	p=0.969	ns	Dunnett
Panel J	eYFP=14	eYFP=14	p=0.358	ns	Signed-Rank
	Control=14	Control=14	p=0.0295	*	Signed-Rank
	cKO=14	cKO=14	p=0.0134	*	Signed-Rank
Panel K	eYFP=14	eYFP=14	p=0.670	ns	Signed-Rank
	Control=14	Control=14	p=0.0203	*	Signed-Rank
	cKO=14	cKO=14	p=0.00854	**	Signed-Rank
Panel N	eYFP=11 Control=12 cKO=15	eYFP=11 Control=12 cKO=15	p=0.0444	*	Kruskal-Wallis
	eYFP vs Control	eYFP=11 Control=12	p=0.0370	*	Tukey's
	eYFP vs cKO	eYFP=11 cKO=15	p=0.535	ns	Tukey's
	Control vs cKO	Control=12 cKO=15	p=0.282	ns	Tukey's

bioRxiv preprint doi: https://doi.org/10.64898/2026.07.24.740623; this version posted July 25, 2026. The copyright holder for this preprint (which was not certified by peer review) is the author/funder, who has granted bioRxiv a license to display the preprint in perpetuity. It is made available under a CC-BY 4.0 International license.

**S Figure 1**		**Animals**	**Sample Size**	**p-values**	**Stars**	**Statistical test**
Panel B		na	na	p=0.0000000153	***	Kruskal-Wallis
		2mM=12 1mM=3	2mM=25 1mM=6	p=0.000573	***	Tukey's
		2mM=12 0.5mM=2	2mM=25 0.5mM=4	p=3.90E-07	***	Tukey's
		1mM=3 0.5mM=2	1mM=6 0.5mM=4	p=0.0657	ns	Tukey's
				p=0.000166	***	Kruskal-Wallis
Panel C		2mM=12 1mM=3	2mM=68 1mM=21	p=0.0191	*	Tukey's
		2mM=12 0.5mM=2	2mM=68 0.5mM=10	p=0.000816	***	Tukey's
		1mM=3 0.5mM=2	1mM=21 0.5mM=10	p=0.492	ns	Tukey's
Panel D		1mM=3	FSCV=6 IPSC=21	p=0.977	ns	Wilcoxon rank-sum
		0.5mM=2	FSCV=4 IPSC=10	p=0.945	ns	Wilcoxon rank-sum
**S Figure 2**						
Panel A		Control=2 cKO=2	Control=23 cKO=24	p=0.292	ns	Wilcoxon rank-sum
		Control=2 cKO=2	Control=23 cKO=24	p=0.219	ns	Wilcoxon rank-sum
Panel B		Control=8 cKO=8	Control=8 cKO=8	p=0.130	ns	Wilcoxon rank-sum
		Control=8 cKO=8	Control=8 cKO=8	p=0.161	ns	Wilcoxon rank-sum
		Control=8 cKO=8	Control=8 cKO=8	p=0.505	ns	Wilcoxon rank-sum
**S Figure 3**						
Panel B		Control=2	Pre=10 Post=10	p=0.000183	***	Wilcoxon rank-sum
		cKO=3	Pre=13 Post=13	p=0.0000165	***	Wilcoxon rank-sum
		vKO=4	Pre=15 Post=15	p=0.00000339	***	Wilcoxon rank-sum
Panel D		Control=2	Pre=5 Post=5	p=0.0079	**	Wilcoxon rank-sum
Panel H		Control=7 CRSIPR=7	Control=7 CRISPR=7	p=0.000583	***	Wilcoxon rank-sum
Panel K		??	??	??	***	student t-test
Panel M		GFP=2 Tetanus=2	na	p=0.00000000104	***	Kruskal-Wallis
		GFP=2	Resp=18 Nresp=22	p=3.10E-07	***	Tukey's
		GFP=2 Tetanus=2	Resp=18 Tet=39	p=3.68E-09	***	Tukey's
		GFP=2 Tetanus=2	Nresp=22 Tet=39	p=0.983	ns	Tukey's
Panel O		Control=3 cKO=3	Control=20 cKO=20	p=0.818	ns	Wilcoxon rank-sum
Panel P		Control= cKO=	Control= cKO=	p=0.1412	ns	F-test
Panel Q		Control= cKO=	Control= cKO=	p=0.4678	ns	student t-test
**S Figure 4**						
Panel D		FSCV=7 ISPC=8	FSCV=15 IPSC=25	p=0.000000175	***	Wilcoxon rank-sum
**S Figure 5**						
Panel C		Veh=3 SCH=3	Veh=9 Sulp=12	p=0.000516	***	Wilcoxon rank-sum
		cKO=5 SCH=2	Veh15= Sulp=11	p=0.0037	**	Wilcoxon rank-sum
		Contrl=3 cKO=5	Control=9 cKO=15	p=0.121	ns	Wilcoxon rank-sum

bioRxiv preprint doi: https://doi.org/10.64898/2026.07.24.740623; this version posted July 25, 2026. The copyright holder for this preprint (which was not certified by peer review) is the author/funder, who has granted bioRxiv a license to display the preprint in perpetuity. It is made available under a CC-BY 4.0 International license.

Panel E		Control=3 cKO=3	Control=13 cKO=21	p=0.0000534	***	Wilcoxon rank-sum
Panel G		Veh=3 SKF=3	Veh=15 SKF=15	p=0.00000339	***	Wilcoxon rank-sum
		Veh=3 SKF=3	Veh=12 SKF=16	p=0.0000115	***	Wilcoxon rank-sum
		Control=3 cKO=3	Control=15 cKO=12	p=0.000278	***	Wilcoxon rank-sum
**S Figure 6**						
Panel A		Control=6 cKO=5	Control=6 cKO=5	p=0.507	ns	Wilcoxon rank-sum
		Control=6 cKO=5	Control=6 cKO=5	p=0.0952	ns	Wilcoxon rank-sum
Panel B		Control=6 cKO=5	Control=6 cKO=5	p= 0.0043	**	Wilcoxon rank-sum
		Control=6 cKO=5	Control=6 cKO=5	p= 0.0043	**	Wilcoxon rank-sum
Panel C		Control=6 cKO=5	Control=6 cKO=5	p=0.537	ns	Wilcoxon rank-sum
		Control=6 cKO=5	Control=6 cKO=5	p=0.0152	*	Wilcoxon rank-sum
Panel D		Control=6 GFP=3	Control=6 GFP=3	p=0.0357	*	Wilcoxon rank-sum
**S Figure 8**						
Panel C		Control=3	Veh=5 SCH=5	p=0.00790	**	Wilcoxon rank-sum
Panel D		Control=3	Veh=12 SCH=5	p=0.00190	**	Wilcoxon rank-sum
Panel E		cKO=3	Veh=6 SCH=6	p=0.00220	**	Wilcoxon rank-sum
Panel F		cKO=3	Veh=12 SCH=6	p=0.000754	***	Wilcoxon rank-sum
**S Figure 9**						
Panel B		na	na	p=1.38E-38	***	Kruskal-Wallis
		cKO=4	Mean=48 Four=48	p=0.000179	***	Tukey's
		cKO=4	Mean=48 Six=46	p=0.0000000207	***	Tukey's
		cKO=4	Mean=48 Eight=34	p=0.0000000207	***	Tukey's
		cKO=4	Mean=48 Ten=25	p=0.0000000207	***	Tukey's
		cKO=4	Mean=48 Twelve=13	p=0.0000000207	***	Tukey's
Panel D		na	na	p=1.7884E-50	***	Kruskal-Wallis
		cKO=4	Mean=48 Four=48	p=0.0184	***	Tukey's
		cKO=4	Mean=48 Six=48	p=0.0000000732	***	Tukey's
		cKO=4	Mean=48 Eight=48	p=0.0000000207	***	Tukey's
		cKO=4	Mean=48 Ten=47	p=0.0000000207	***	Tukey's
		cKO=4	Mean=48 Twelve=45	p=0.0000000207	***	Tukey's
**S Figure 10**						
Panel B		Control=4 cKO=4	Control=70 cKO=44	p=2.01e-16	***	Wilcoxon rank-sum
Panel C		Control=4 cKO=4	Control=70 cKO=44	p=1.05E-14	***	Wilcoxon rank-sum
Panel D		cKO=4	Mean=44 Peak=44	p=6.80e-16	***	Wilcoxon rank-sum
Panel E		Control=4 cKO=4	Control=70 cKO=49	p=6.58e-15	***	Wilcoxon rank-sum
Panel F		Control=4 cKO=4	Control=70 cKO=49	p= 2.73e-15	***	Wilcoxon rank-sum

bioRxiv preprint doi: https://doi.org/10.64898/2026.07.24.740623; this version posted July 25, 2026. The copyright holder for this preprint (which was not certified by peer review) is the author/funder, who has granted bioRxiv a license to display the preprint in perpetuity. It is made available under a CC-BY 4.0 International license.

Panel G		cKO=4	Mean=49 Peak=49	p=1.93e-17	***	Wilcoxon rank-sum
**S Figure 11**						
Panel D		Control=3 cKO=3	Control=23 cKO=24	p=0.663	ns	Wilcoxon rank-sum
		Control=3 cKO=3	Control=22 cKO=24	p=0.558	ns	Wilcoxon rank-sum
Panel E		Control=3 cKO=3	Control=23 cKO=23	p=0.5679	ns	Wilcoxon rank-sum
		Control=3 cKO=3	Control=23 cKO=23	p=0.7584	ns	Wilcoxon rank-sum
**S Figure 12**						
Panel B		Control=16 6OHDA=8	Control=16 6OHDA=8	p=0.000101	***	Wilcoxon rank-sum
		Control=16 6OHDA=8	Control=16 6OHDA=8	p=0.000101	***	Wilcoxon rank-sum
		Control=16 6OHDA=8	Control=16 6OHDA=8	p=0.00240	**	Wilcoxon rank-sum
Panel E		Control=8 6OHDA=14	Control=8 6OHDA=14	p=0.0046	**	Wilcoxon rank-sum
Panel F		Control=8 6OHDA=14	Control=8 6OHDA=14	p=0.609	ns	Wilcoxon rank-sum
Panel G		Cont.=14 cO=17 vKO=16 Res=15	Cont.=14 cO=17 vKO=16 Res=15	p=0.433	ns	Kruskal-Wallis
Panel J		na	na	p=0.0209	*	Kruskal-Wallis
		eYFP=11 Control=12	eYFP=11 Control=12	p=0.0256	*	Tukey's
		eYFP=11 cKO=14	eYFP=11 cKO=14	p=0.841	ns	Tukey's
		Control=12 cKO=14	Control=12 cKO=14	p=0.0745	ns	Tukey's
Panel L		eYFP=14	eYFP=14	p=0.358	ns	Signed-Rank
		eYFP=14	eYFP=14	p=0.670	ns	Signed-Rank
		Control=14	Control=14	p=0.0203	*	Signed-Rank
		Control=14	Control=14	p=0.0295	*	Signed-Rank
		cKO=14	cKO=14	p=0.0134	*	Signed-Rank
		cKO=14	cKO=14	p=0.00854	**	Signed-Rank
Panel M		eYFP=14 Control=14 cKO=14	eYFP=14 Control=14 cKO=14	p=0.0828	ns	Kruskal-Wallis
		eYFP=14 Control=14 cKO=14	eYFP=14 Control=14 cKO=14	p=0.677	ns	Kruskal-Wallis
		eYFP=14 Control=14 cKO=14	eYFP=14 Control=14 cKO=14	p=0.315	ns	Kruskal-Wallis
